# Novel technique of vulvo-vaginal rejuvenation by lipofilling and injection of combined platelet-rich-plasma and hyaluronic acid: a case-report

**DOI:** 10.1186/s40064-016-2840-y

**Published:** 2016-07-26

**Authors:** Paola Aguilar, Barbara Hersant, Mounia SidAhmed-Mezi, Romain Bosc, Luciano Vidal, Jean Paul Meningaud

**Affiliations:** Department of Plastic, Reconstructive and Aesthetic Surgery, Henri Mondor Hospital, 51 Avenue du Maréchal de Lattre de Tassigny, 94010 Créteil, France

**Keywords:** Vaginoplasty, Perineal rejuvenation, Lipofilling, Platelet-rich plasma, Hyaluronic acid, Sexual dysfunction

## Abstract

**Background:**

To describe a new surgical procedure and its results: the vulvo-vaginal rejuvenation by lipofilling and an injection of combined platelet-rich-plasma (PRP) and hyaluronic acid (HA). Sexual life for women is affected by the effect of aging and by post partum traumatism. There are no standard non-invasive treatments to offer to improve the trophic and dimensional alterations of the vulvo-vaginal area. The surgical procedure consists in a vaginoplasty by lipofilling of the posterior vaginal wall far from the vascular axes and with an injection of an injection of combined PRP and HA subcutaneously in the perineum. To illustrate the technique and evaluate its results, we present the case of a 39 year-old-female with history of episiotomy presented that vaginal laxity resistant to physical therapy. To assess the results regarding the sexual quality of life we used the modified Stabbatsberg self-rating scale.

**Findings:**

There were no intra-operative complications with this simple procedure. During follow-up we observed an improvement in the modified Stabbatsberg scale and a vulvo-perineal rejuvenation by improving the vaginal trophicity and restoring a normal vaginal caliber. No post-operative complications occurred.

**Conclusions:**

Vulvo-vaginal rejuvenation lipofilling and an injection of combined platelet-rich-plasma and hyaluronic acid is a minimally invasive technique that is safe and easy to perform. Further studies are necessary to assess more thoroughly the effectiveness and safety of this procedure and assess medium and long term results.

## Background

Aging and obstetric perineal trauma can be the cause of trophicity and dimensional problems such as dyspareunia and vaginal laxity and leading to postpartum sexual dysfunction (Signorello et al. 2001). This frequent condition has a negative impact on women’s quality of life and sexual intercourse (Pauls et al. 2012).

Vaginal laxity mostly affects women that had no access to perineal rehabilitation (Harvey 2003), which is known to lessen the occurrence of these symptoms when performed early after vaginal delivery. Colporrhaphy is a well-established surgical approach to treat vaginal laxity. And there are few non-invasive treatment options as well such as HA injection or fat transplantation alone.

Episiotomy sequelae such as painful scar responsible of dyspareunia, retractile and unaesthetic scaring are frequent. Studies (Eppley et al. 2006) have shown that platelet-rich-plasma (PRP) promotes the wound healing process by releasing growth factors. Furthermore, hyaloronic acid (HA) potentiates this effect, enhances the hydratation of tissues and the adjunction of the HA to the PRP solution facilitate its use by turning it into a more viscous solution.

In this article we describe our vaginoplasty technique which combines a vaginal lipofilling and a perineal rejuvenation consisting in injecting a solution of PRP and HA. We illustrate this technique with a clinical case.

## Methods

### Surgical technique

The patient gave her consent for the use of her personal and medical information, and accompanying photos in the publication of this case report.

The procedure is performed under general anesthesia. The patient is installed in a gynecologic position.

The procedure involved several steps: the isolation of PRP, the mixing of PRP with HA, the harvest, purification and injection of fat cells in the posterior vaginal wall and the injection of the PRP-HA preparation in the perineal raphe, in the vestibular fossa and in the labius minus and majus is they are atrophic.

The technical elements of each step are as follows:

For the PRP-HA preparation we use the RegenKit^®^-BCT Cellular Matrix kit. The peripheral blood is withdrawn from the patient and collected into three single use sterile tubes (4 mL per tube). Each tube contained a gel of HA, and inert cell-selector gel, a liquid anticoagulant and allowed the preparation of 4 mL of PRP-HA mixture (2 mL of PRP for 2 mL of HA).

The tubes are centrifuged at 1500*g* for 5 min.

After centrifugation, the blood is fractionated: the red blood cells are trapped under the gel and cellular elements settle on the surface of the gel. The red blood cells depletion is the 99.7 %. The platelet concentration compared to the blood flow is to 1.6 %.

**Fig. 1:**
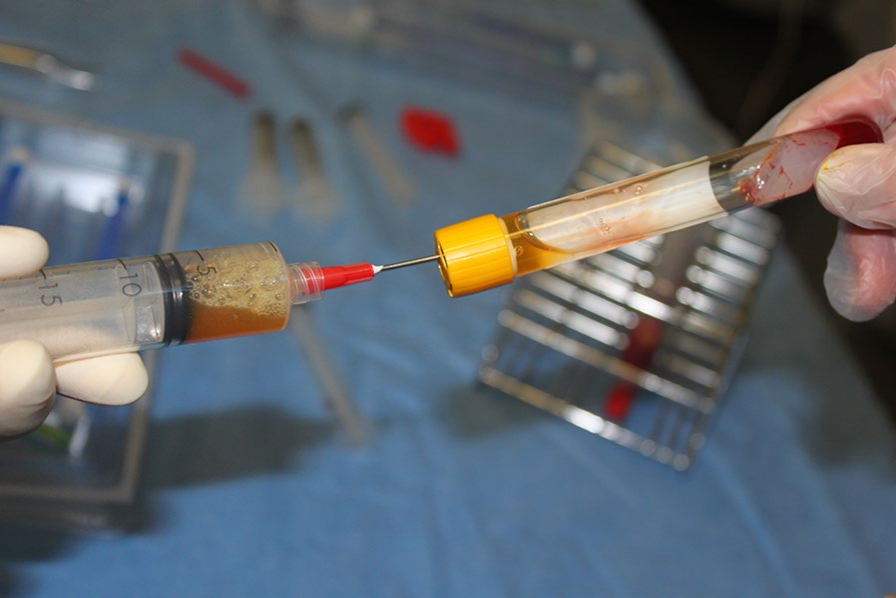
Retreaving the PRP-HA mixture must be done carefully and in sterile conditions

HA is positioned on top of the PRP. After manual homogenization of the PRP with the HA, the mixture is withdrawn with an external sterile syringe and needle (Fig. 1). Concomitantly the fat is harvested according Coleman’s technique (Cihantimur and Herold 2013) in the lower abdomen area with a cannula of 3 mm, a 10 mL syringe (without any previous local infiltration) and was then centrifuged at 1500*g* for 1 min.

The sub-mucosal injection in the posterior vaginal wall and the sub-dermal injection in the perineum are performed under digital control (one finger in the rectum).

The important point of this step is to avoid the principal vascular axes of the vaginal to prevent haematomas and emboli. The safest area to inject is the posterior wall of the vagina. The injection of the fat graft has to be homogenous.

The volume of fat graft injected and of the mixture of PRP + HA varies for every patient. Must be taken into account the quantity of available fat donor sites, the tolerance of the skin and the mucosa while injecting. We advice not to inject more than 30 cc of fat graft because of the increase risk of fat necrosis and infection in this area.

An antibiotic prophylaxis by amoxicilline-acid-clavulanic was administrated for 48 h.

The total duration of the procedure was 30 min.

The price of the RegenKit^®^-BCT Cellular Matrix kit is off 210 euros.

## Results

It is the case of a 39-year-old primiparous woman referred to our department for sexual dysfunction. When she was 23, she had her only child by vaginal delivery in Africa. An episiotomy was performed during the delivery; no obstetric forceps were used to extract the baby. No perineal immediate postpartum physiotherapy was performed.

The first sexual dysfunction symptoms started 7 years after birthdate when she resumed her sexual vaginal activity. Her symptoms dealt with vaginal laxity and included: flatus incontinence, an unpleasant feeling of a too wide vagina, sexual dysfunction reported by partner complaints of not feeling her vaginal walls, the overall patient’s sexual dissatisfaction and her specific conviction of not being able to reach orgasms. She did not notice any improvement of her symptoms after an intensive perineal physiotherapy treatment (40 sessions) that she started 7 years after birthdate, leading to low self-confidence and psychological disturbance. She had no history of urinary or anal incontinence. Her comorbidities were a double homozygote SC drepanocytosis, an umbilical hernia repair, a cholecystectomy, and an abdominal dermolipectomy. She did not take any medication or hormonal contraception. Clinical examination showed a vaginal laxity with preserved vaginal tonicity, a retractile episiotomy scar, a slightly atrophic vaginal mucosa and a pelvic floor muscle laxity with a diastasis of the anus elevators. We did not find out any vaginal tissue defect, nor prolapse.

**Fig. 2:**
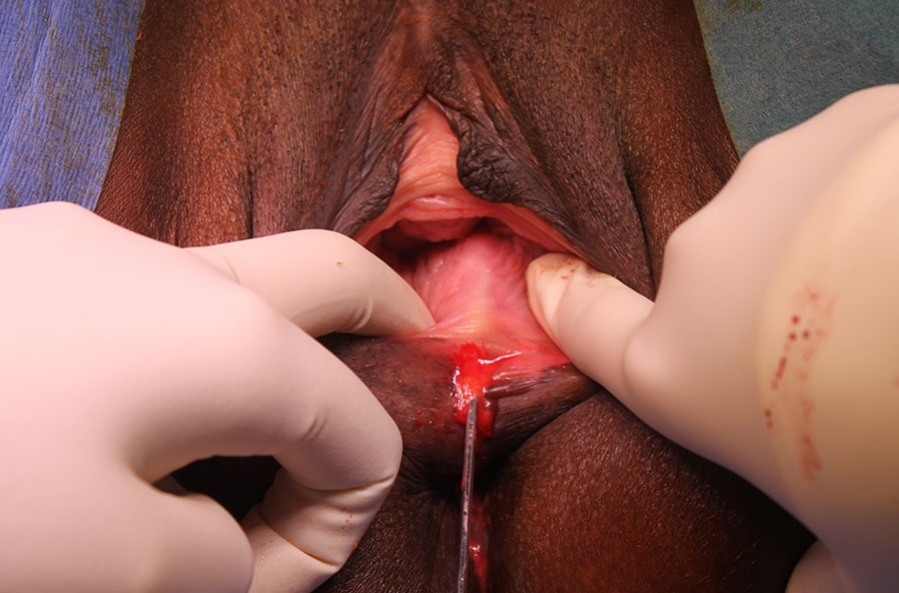
The lipofilling of the posterior vaginal wall must be performed under digital control to stay in the sub mucosa and avoid the risk of rectal injury

**Fig. 3:**
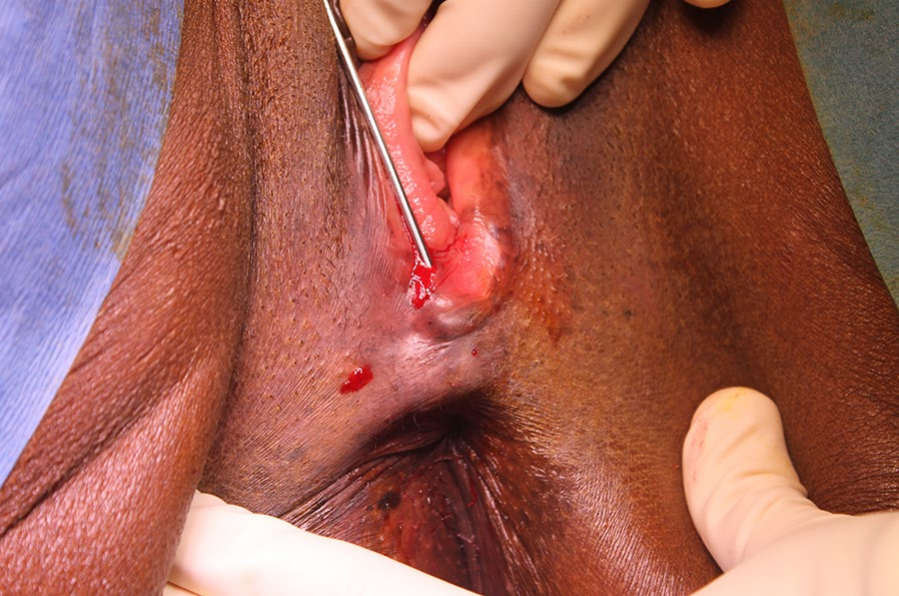
The subcutaneous injection of the PRP-HA solution in the perineum is done after fasciotomy

During the intervention, we injected sixteen milliliters of purified fat cells into two linear antero-posterior tractus in the posterior vaginal wall and ten milliliters of the PRP-HA solution in the perineum focusing in the episiotomy scar (Figs. 2, 3).

**Fig. 4:**
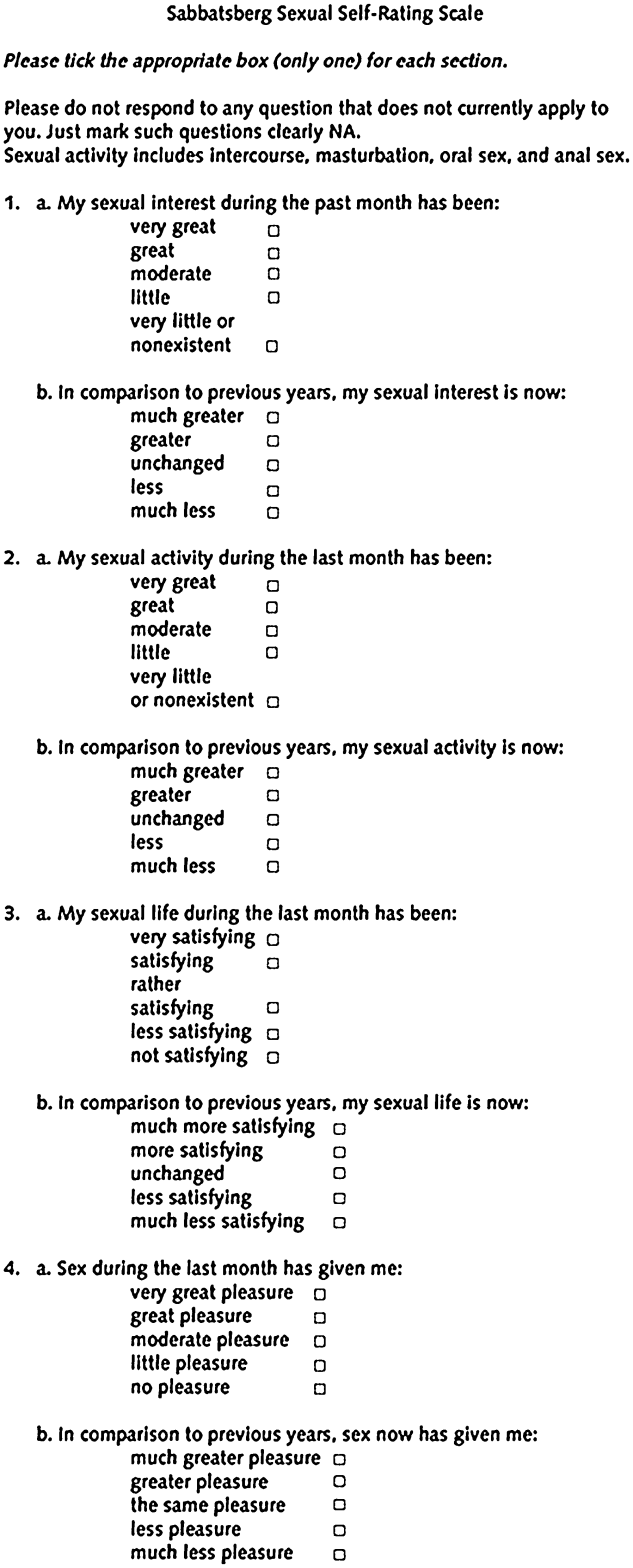
Sabbatsberg sexual self-rating scale

We used the standard and validated Stabbatsberg sexual self rating scale (Garratt et al. 1995) (Fig. 4) to asses the repercussions of this affection in her sexual life. It is a standardized self-administered questionnaire that assesses four items: sexual interest, sexual activity, sexual life and pleasure during sex. We translated it to the patient’s native language (French) and determinated a score for each item. A high score indicates a satisfactory sexual life and a low score a deteriorated one. The pre-operatory score of the patient was 15/40, indicating a major negative impact on her sexual life.

No atrophy or hardening was found, nor any signs of infection.

The patient was allowed to resume her sexual activity at 1-month follow-up.

At 3-month follow up, the symptoms improved significantly. The patient was pleased with the result and the improvement of her sexual life. Flatus incontinence disappeared completely. Clinical examination showed a tighter vagina with a diminished vaginal caliber, an enhancement of vaginal mucosa trophicity, and an episiotomy scar softer and more hydrated. Her Stabbatsberg scale score was 30/40 i.e. 100 % of improvement.

## Discussion

Very little literature is currently available regarding the minimally invasive surgical options for vulvo-vaginal rejuvenation and for treatment of sexual dysfunction.

Vaginal surgical tightening has been proven to have good aesthetic results and a patient satisfaction rate by Cihantimur and Herold (2013), but with the invasive approach of the hydrodissection of the vaginal mucosa, excision of portion of the posterior mucosa from vaginal fornices via radiofrequency surgery and suture of the vaginal edges. The method we described has the advantage to be a mini-invasive surgical technique.

No documented cases using the combination of theses three techniques are reported in the PubMed database.

Lipofilling is a popular reconstructive technique that uses an extemporaneous, autologous material. Fat grafting using the Coleman’s technique (Coleman 2004) is simple, safe, reliable and time-effective, with a initial depertition of 30 % of the fat transplant and stabilization of the results at 3 months, which is why we decided to evaluate our results at 3 months after the surgery. There are few cases describing the use of this technique in the perineal area, Zelitz et al. (2013) reported good results using fat grafting for the reconstruction of vaginal wall defects. Herold et al (2015) described a G-spot augmentation by injection of autologous fat in the anterior vaginal wall without any postoperative complications. Concerning our case, we were meticulous and careful with the fat harvest because the patient had poor donor sites. Subsequently, we think that we might have injected a major amount of fat graft.

Hyaluronic acid is widely used in cosmetic procedures; this technique is mostly innocuous but several cases of non-thrombotic pulmonary embolism (NTP) have been reported Park et al. (2010).

Hyaluronic acid is a dermal filler used to cure facial wrinkles but its use in the vaginal sphere is very recent and poorly documented.

In the case reported by Park et al. (2010) NTP was believed to have occurred in the systemic circulation, because hyaluronic acid was injected to the anterior wall of the vagina were there is an extensive venous plexus. In our case we avoided injecting this highly vascularized area.

A recent study by Chen et al. (2013) showed the efficiency and safety of HA vaginal gel to treat vaginal dryness without local or general complication. Regarding this results we hypothesize that injecting HA in the vaginal mucosa may directly enhance its trophicity, but further studies need to be carried out. Hyaluronic acid plays an important role in tissue regeneration processes by facilitating the influx of many different types of cell into an area of damage. Bourguignon (2014) reported that HA interact with cell surface receptors such as CD44 inducing cell–cell adhesions, cell substrate adhesions, cell proliferation and cell migration.

PRP contains growth factors as PDGF, TGF-B, IGF, EGF, VEGF released by platelets that have an important role in inflammation reduction, angiogenesis stimulation, and collagen III synthesis (Eppley et al. 2006). Several studies have shown a beneficial effect of the PRP on wound healing. Hua et al (2012) reported promising results with PRP in the gynecological sphere to cure symptomatic cervical ectopy. We believe that mixing PRP with hyaluronic acid potentialize its effect by facilitating the integration of the filler in surrounding tissues, and easing the healing process. Nevertheless as Kushida et al. (2014) showed in their comparative study of eight PRP separation system (not including any Regen Lab kit), they vary widely in terms of platelet, white blood cell, red blood cell, growth factor, platelet-derived growth factor, transforming growth factor beta-1 and vascular endothelial growth factor. The total dose of platelets for 2 mL of PRP prepared with the RegenKit^®^-BCT Cellular Matrix kit was of 0.9 billion and the obtained concentration of growth factor is as follows: TGF-beta: 67.37 ng/mL, PFGF-AB: 12.71 ng/mL, bFGF: 0,06 ng/mL, IGF: 82.22 ng/mL, VEGF: 0.87 ng/mL, EGF: 0.93 ng/mL (data on file).

Our results are encouraging. The benefit of this procedure seems to be on the mechanical aspect of the sexual dysfunction as incontinence flatus. The improvement of global sexual life quality is supported by a simple, acceptable, valid, and reliable scale.

We assume that anonymity in that kind of questionnaire will be more objective however we do not believe that the patient retained information; she was very cooperative. Concerning the absence of vaginal orgasm, we have to bear in mind the high subjectivity of this matter. We did not take into account the other factors that might influence sexual life quality as depression, fatigue, partner expertise, etc.

## Conclusion

The vaginoplasty with lipofilling technique is a simple mini-invasive method that is not currently described in the literature. We wanted to share our experience in that domain and illustrate its efficiency and safety.

We believe that the combined injection of PRP and HA technique should be considered part of the therapeutic armamentarium used for perineal rejuvenation as well.

Although we didn’t test for it we believe this technique will also other perineal aging consequences as vaginal dryness and vulvary pain. It is important to emphasize that a multidisciplinary approach is essential to asses before surgery the anatomical implication of the sexual dysfunction that will respond to surgical treatment and the psychosocial, relational and sexological aspects that need specific treatment.

We recognize the limitations of a single-subject presentation, but our results encouraged us to share our experience without any delay. A multicenter clinical trial is necessary to described more thoroughly the effectiveness and safety of this technique and the stability of the results in the medium and long term, we are at the disposal of any interested team.
